# Altered Cerebellar Short-Term Plasticity but No Change in Postsynaptic AMPA-Type Glutamate Receptors in a Mouse Model of Juvenile Batten Disease

**DOI:** 10.1523/ENEURO.0387-17.2018

**Published:** 2018-05-17

**Authors:** Dorota Studniarczyk, Elizabeth L. Needham, Hannah M. Mitchison, Mark Farrant, Stuart G. Cull-Candy

**Affiliations:** 1 Department of Neuroscience, Physiology and Pharmacology, University College London, London WC1E 6BT, United Kingdom; 2 UCL Great Ormond Street Institute of Child Health, University College London, London WC1N 1EH, United Kingdom

**Keywords:** AMPA receptors, Batten disease, cerebellum, CLN3, EPSCs, short-term plasticity

## Abstract

Juvenile Batten disease is the most common progressive neurodegenerative disorder of childhood. It is associated with mutations in the *CLN3* gene, causing loss of function of CLN3 protein and degeneration of cerebellar and retinal neurons. It has been proposed that changes in granule cell AMPA-type glutamate receptors (AMPARs) contribute to the cerebellar dysfunction. In this study, we compared AMPAR properties and synaptic transmission in cerebellar granule cells from wild-type and *Cln3* knock-out mice. In *Cln3*^Δ*ex1–6*^ cells, the amplitude of AMPA-evoked whole-cell currents was unchanged. Similarly, we found no change in the amplitude, kinetics, or rectification of synaptic currents evoked by individual quanta, or in their underlying single-channel conductance. We found no change in cerebellar expression of GluA2 or GluA4 protein. By contrast, we observed a reduced number of quantal events following mossy-fiber stimulation in Sr^2+^, altered short-term plasticity in conditions of reduced extracellular Ca^2+^, and reduced mossy fiber vesicle number. Thus, while our results suggest early presynaptic changes in the *Cln3* ^Δ^*^ex1–6^* mouse model of juvenile Batten disease, they reveal no evidence for altered postsynaptic AMPARs.

## Significance Statement

Juvenile Batten disease is an inherited lysosomal storage disorder that affects children and leads to premature death. Caused by mutations in the *CLN3* gene, it results in a loss of CLN3 protein and neuronal degeneration. It has been proposed that changes in granule cell AMPA-type glutamate receptors (AMPARs) contribute to cerebellar dysfunction. Here, we show that the properties of postsynaptic AMPA receptors in granule cells from juvenile *Cln3* ^Δ*ex1–6*^ mice are unaltered. Instead, loss of CLN3 protein leads to early presynaptic changes and altered short-term plasticity.

## Introduction

Batten disease is the collective term for a group of rare inherited neurodegenerative disorders, the neuronal ceroid lipofuscinoses (NCLs). These result from mutations in one of 14 ceroid-lipofuscinosis, neuronal type (CLN) genes (Cotman et al., 2013; Mole and Cotman, 2015; Nita et al., 2016), the majority of which encode soluble lysosomal enzymes or lysosome-associated transmembrane proteins (Cárcel-Trullols et al., 2015). The most common NCL is juvenile CLN3 disease or juvenile Batten disease (Williams and Mole, 2012). Children with this condition first exhibit symptoms at four to seven years of age, suffer loss of vision, seizures, progressive motor and cognitive decline, and die prematurely in late adolescence (Munroe et al., 1997; Haltia, 2003).

Juvenile Batten disease is caused by mutations in the *CLN3* gene, commonly a 1-kb deletion encompassing exons 7 and 8, that result in the loss of full-length CLN3 protein (The International Batten Disease Consortium, 1995; Munroe et al., 1997; Kitzmüller et al., 2008). Like other NCLs, juvenile Batten disease is considered a lysosomal storage disorder and is characterized by the accumulation within lysosomes of autofluorescent lipopigments (lipofuscin-like ceroid; Seehafer and Pearce, 2006). Although the precise function of CLN3 remains unresolved, it has been implicated in multiple cellular phenomena, including endocytosis and endocytic trafficking, lysosmal pH regulation, autophagy, proliferation, cell-cycle control, and apoptosis (Cárcel-Trullols et al., 2015).

Cerebellar atrophy is a feature of juvenile Batten disease (Nardocci et al., 1995; Autti et al., 1996) and likely contributes to the eventual motor deficits (Raininko et al., 1990). Likewise, in mouse models of the disease, there are degenerative changes and neuronal loss in the cerebellum, seen most clearly in CLN3 knock-out animals (designated *Cln3* ^Δ*ex1–6*^ or *Cln3^–/–^*; Kovács et al., 2006; Weimer et al., 2009) but also evident in mice with knock-in of the most common human 1-kb deletion mutation (*Cln3* ^Δ^*^ex7/8^*; Cotman et al., 2002).

Several studies have provided evidence of a change in neuronal AMPA-type glutamate receptors (AMPARs) in juvenile Batten disease. Thus, in *Cln3* ^Δ*ex1–6*^ and *Cln3* ^Δ^*^ex7/8^* mice, cerebellar granule cells – neurons in the cerebellum that relay multisensory and motor-related information from mossy fibers to Purkinje cells (Eccles et al., 1967; Huang et al., 2013; Chabrol et al., 2015) – are reported to exhibit increased susceptibility to excitotoxic damage following activation of AMPARs (Kovács et al., 2006; Finn et al., 2011). These receptors, mediate a majority of fast excitatory transmission in the brain, and function as homo- or hetero-tetrameric assemblies of pore-forming subunits (GluA1-4; Traynelis et al., 2010). Although most AMPARs in the central nervous system contain the edited GluA2(R) subunit, and are thus calcium impermeable (CI-AMPARs), those lacking GluA2 constitute a widely distributed subtype of calcium permeable AMPARs (CP-AMPARs; Burnashev et al., 1992; Geiger et al., 1995; Cull-Candy et al., 2006).

Excess influx of Ca^2+^ through CP-AMPARs appears to be a feature common to several neurodegenerative disorders, including stroke, motor neuron disease, and hypoxic ischemic white matter damage (Follett et al., 2000; Kawahara and Kwak, 2005; Noh et al., 2005; Van Den Bosch et al., 2006; Corona and Tapia, 2007). Increased AMPAR-mediated excitotoxicity in *Cln3* ^Δ*ex1–6*^ mice has been suggested to reflect altered AMPAR trafficking, an increase in CP-AMPAR number and enhanced AMPAR function (Kovács et al., 2006). However, recent experiments have described an increase in GluA2 protein in the cerebellum of *Cln3* ^Δ*ex1–6*^ mice (Kovács et al., 2015), a change which is more usually associated with increased prevalence of CI-AMPAR subtypes.

Here, we have compared AMPAR properties and excitatory synaptic transmission in cerebellar granule cells from wild-type and *Cln3* ^Δ^*^ex1–6^*mice. Our results suggest that loss of CLN3 results in altered mossy-fiber presynaptic behavior but no alteration in postsynaptic AMPAR function and no increase in CP-AMPAR prevalence.

## Materials and Methods

### Animals

We used wild-type C57BL/6J mice and *Cln3* knock-out mice (*Cln3* ^Δ*ex1–6*^) on a C57BL/6J background. *Cln3* ^Δ*ex1–6*^ mice were generated via targeted disruption of the *Cln3* gene involving the deletion of exons 2–6 and most of exon 1 via replacement with a neomycin resistance gene that was transcribed in reverse orientation from a mouse PGK promoter (Mitchison et al., 1999). Both male and female mice were used. All procedures for the care and treatment of mice were in accordance with the Animals (Scientific Procedures) Act 1986.

### Western blotting

Cerebellar tissue was homogenized in RIPA lysis buffer with proteinase inhibitors (Roche). Protein extracts were boiled for 5 min at 95°C before loading onto 5–10% gradient gels (50 μg of protein sample per lane). Gels were electrotransferred to a 0.2-μm nitrocellulose membrane (GE Healthcare). Blots were blocked in 4% milk (wt/vol) in PBS-Tween 20 solution for 1 h, then incubated at 4°C overnight with one of the following antibodies: anti-GluA2 (mouse, Millipore MAB397, 1:500), anti-GluA4 (rabbit, Millipore AB1508, 1:200), anti-cofilin (rabbit, Abcam ab42824, 1:10,000). Transferred proteins were detected with appropriate horseradish peroxide-conjugated (HRP) secondary antibodies: goat anti-mouse IgG-HRP (Santa Cruz sc-2005, 1:2000) or goat anti-rabbit IgG-HRP (Santa Cruz sc-2030, 1:2000), reacted with chemiluminescent ECL substrate (Thermo Scientific Pierce), and visualized by ChemiDoc MP System (Bio-Rad Laboratories). Band intensities of GluA2 and GluA4 were normalized to the respective cofilin bands or to the total protein determined by Ponceau S staining of the membranes (Image Lab 5.2, Bio-Rad Laboratories).

### Dissociated cerebellar cultures

Cultures of dissociated cerebellar neurons were prepared from postnatal day (P)5–P7 mice. Briefly, after decapitation, the cerebella were removed, cut into small pieces and trypsinized at 37°C. Mechanically dissociated cells were plated on poly-l-lysine-coated (Sigma) glass coverslips, at a density of 2.1 × 10^5^ cells per coverslip. Cells were maintained in a humidified atmosphere at 37°C (5% CO_2_) in basal medium Eagle (BME) supplemented with 10% fetal bovine serum (FCS; v/v), 2 mM l-glutamine, and 100 mg ml^−1^ gentamicin (all Gibco). Cells were maintained in “high K^+^” (25 mM KCl) to promote synaptic maturation. Cytosine arabinoside (10 μM; Sigma) was added 24 h after plating to inhibit glial proliferation. In most cases, wild-type and *Cln3* ^Δ*ex1–6*^ cultures were prepared concurrently and examined in interleaved recordings after 7–13 d.

### Electrophysiology of cultured granule cells

Cells, identified according to previously described criteria (Cull-Candy et al., 1988), were viewed using a fixed-stage microscope (Zeiss Axioskop FS1 or Olympus BX51WI) and perfused at a rate of 1.5–2 ml min^−1^ (2-ml bath volume)_._ The extracellular solution contained 145 mM NaCl, 2.5 mM KCl, 1 mM CaCl_2_, 1 mM MgCl_2_, 10 mM glucose, and 10 mM HEPES (adjusted to pH 7.3 with NaOH). Pipettes for whole-cell recording were pulled from thick-walled borosilicate glass (1.5 mm o.d., 0.86 mm i.d., Harvard Apparatus), coated with Sylgard resin (Dow Corning 184) and fire-polished to a final resistance of ∼5–8 MΩ. Pipettes were filled with a solution containing 145 mM CsCl, 2.5 mM NaCl, 1 mM Cs-EGTA, 4 mM MgATP, and 10 mM HEPES (adjusted to pH 7.3 with CsOH). Spermine tetrahydrochloride (500 μM, Sigma) was added to this intracellular solution immediately before each recording session.

Currents were recorded at 22–26°C using an Axopatch 1D or Axopatch 200B amplifier and acquired using pClamp10 and a Digidata 1200 interface (Molecular Devices). Series resistance and input capacitance were read directly from the amplifier settings used to minimize the current responses to 5-mV hyperpolarizing voltage steps; values were 6.3 ± 0.4 pF for wild-type versus 5.9 ± 0.5 pF for *Cln3* ^Δ*ex1–6*^ (*n* = 34 and 42; W = 861.5, *p* = 0.12 Wicoxon rank sum test) and 25.2 ± 0.8 versus 27.3 ± 1.2 MΩ (W = 604.5, *p* = 0.25 Wilcoxon rank sum test). Whole-cell current−voltage (*I-V*) relationships were generated by ramping membrane potential from –90 to +60 mV in the presence of 20 μM s-AMPA and 10 μM cyclothiazide (Ascent Scientific) applied by gravity-fed bath perfusion. Ramps were delivered once currents had reached steady-state amplitude. Records were filtered at 2 kHz and sampled at 5 kHz. The rectification index (RI) was calculated as the ratio of slope conductance in positive (+20 to +40 mV) and negative (–40 to –20 mV) limbs of the *I-V*.

### mEPSCs in cultured granule cells

Miniature EPSCs (mEPSCs) were recorded at –60 mV after blocking voltage-gated sodium channels, NMDA-, GABA_A_-, and glycine receptors by adding 1 μM tetrodotoxin (TTX), 20 μM d-AP5, 20 μM SR-95531, and 1 μM strychnine (Ascent Scientific). Before mEPSC recording, the cells were briefly exposed (2–3 min) to 200 μM LaCl_3_ to increase mEPSC frequency (Chung et al., 2008). The signal was filtered at 2 kHz and sampled at 20 kHz. Event detection was performed using amplitude threshold crossing (Igor Pro 5, Wavemetrics Inc; NeuroMatic 2.02, www.neuromatic.thinkrandom.com), with the threshold (typically ∼5 pA) set to 3× the baseline current variance. The rectification index (RI_CM_) was calculated by dividing the mean mEPSC peak conductance calculated using all events detected at +60 mV and a matching number of the largest events at –60 mV. For fluctuation analysis (see paragraph below) and kinetic analysis, only events that exhibited a monotonic rise and an uncontaminated decay were included. Such events were aligned on their rising phase before averaging. The decay of the averaged mEPSC was fitted with a double exponential, and the weighted time constant of decay (τ_w, decay_) calculated as the sum of the fast and slow time constants weighted by their fractional amplitudes. In some cases, mEPSCs were adequately fit with single exponentials.

Peak-scaled non-stationary fluctuation analysis (ps-NSFA) was used to estimate the weighted mean single-channel conductance of synaptic receptors (Traynelis et al., 1993; Hartveit and Veruki, 2007). Each mEPSC was divided into 30 bins of equal amplitude, and, within each bin, the variance of the mEPSC about the scaled average was computed. The variance was plotted against the mean current value, and the weighted mean single-channel current was estimated by fitting the full parabolic relationship with the equation:

$$\sigma_{\text{PS}}^{2}=i\,\overset{¯}{I}-{\overset{¯}{I}}^{\;2}/N_{\text{p }}+\sigma_{\text{B}}^{2}$$

where σ^2^_PS_ is the peak-scaled variance, *I¯* is the mean current, *i* is the weighted mean single-channel current, *N*_p_ is the number of channels open at the peak of the EPSC, and σ^2^_B_ is the background variance. The weighted mean chord conductance for each cell was calculated assuming a reversal of 0 mV.

### Acute cerebellar slices

Mice (P10–P15) were anesthetized with isoflurane and decapitated. After brain dissection, 250-μm-thick sagittal slices were cut in an ice-cold oxygenated solution (85 mM NaCl, 2.5 mM KCl, 0.5 mM CaCl_2_, 4 mM MgCl_2_, 25 mM NaHCO_3_, 1.25 mM NaH_2_PO_4_, 64 mM sucrose, and 25 mM glucose; pH 7.3 when bubbled with 95% O_2_ and 5% CO_2_), using a vibratome (Microm HM 650 V or Campden 7000smz). To prevent NMDAR-mediated cell damage 20 μM d-AP5 (Tocris Bioscience) was included. Slices were stored in the same solution at 35°C for 30 min and then transferred into recording “external” solution at 23–26°C (125 mM NaCl, 2.5 mM KCl, 2 mM CaCl_2_, 1 mM MgCl_2_, 25 mM NaHCO_3_, 1.25 mM NaH_2_PO_4_, and 25 mM glucose; pH 7.3 when bubbled with 95% O_2_/5% CO_2_).

### Slice electrophysiology

Slices were viewed using a fixed stage upright microscope (Olympus BX 51WI with infrared differential interference contrast or oblique illumination) and recordings were made from visually identified neurons in the internal granule cell layer (Kaneda et al., 1995). To block NMDA and GABA_A_ receptors, 20 μM d-APV and 20 μM SR-95531 (Ascent Scientific) were added. The internal solution contained 128 mM CsCl, 10 mM HEPES, 10 mM EGTA, 2 mM Mg_2_ATP, 0.5 mM CaCl_2_, 2mM NaCl, 5 mM TEA, 1 mM *N*-(2,6-dimethylphenylcarbamoylmethyl) triethylammonium bromide (QX-314), and 0.1 mM spermine tetrahydrochloride (pH 7.3 with CsOH). Currents were recorded using an Axopatch 200B amplifier, filtered at 2 kHz and digitized at 20 kHz (pClamp 10.2 Molecular Devices or Igor Pro 5 with NeuroMatic). All currents were recorded at room temperature, with the exception of minimally evoked EPSCs (meEPSCs; see ‘Quanta and evoked EPSCs’ below). Series resistance and input capacitance were read directly from the amplifier settings used to minimize the current responses to 5-mV hyperpolarizing voltage steps. Series resistance was compensated (up to 75%). Measured values at room temperature were 3.9 ± 0.3 pF for wild type versus 3.6 ± 0.3 pF for *Cln3* ^Δ*ex1–6*^ (*n* = 11 and 12; W = 76.5, *p* = 0.54 Wilcoxon rank sum test) and 10.0 ± 0.5 versus 12.4 ± 1.0 MΩ (W = 40.5, *p* = 0.12 Wilcoxon rank sum test).

### Quantal and evoked EPSCs

To record quantal EPSCs (qEPSCs), the standard extracellular solution was replaced with a Ca^2+^-free solution containing 5 mM SrCl_2_ (Goda and Stevens, 1994; Abdul-Ghani et al., 1996). Mossy fibers were stimulated (0.5 Hz) using a concentric bipolar tungsten electrode placed in the white matter tract (Digitimer DS/2A constant voltage stimulator; 100 V/200 μs). Events were detected using amplitude threshold crossing, with the threshold (typically ∼5 pA) set according to the baseline current variance. To avoid the inclusion of multiquantal events, only qEPSCs occurring >10 ms after the mossy fiber stimulus were included. When analyzing event frequency, any qEPSC with a distinct peak was included. When analyzing qEPSC amplitude, all events with a monotonic rise were included, irrespective of overlapping decays. For kinetic analysis, only events with a monotonic rise and uncontaminated decay were included; they were aligned on their rising phase before averaging. The decay of the averaged qEPSC was fitted with a double exponential, and the weighted time constant of decay (τ_w, decay_) calculated.

To record eEPSCs, mossy fibers were stimulated (0.5 Hz) using a concentric bipolar tungsten electrode placed in the white matter tract (Digitimer DS/2A constant voltage stimulator). Pairs of eEPSCs were recorded at room temperature with an extracellular solution containing 2 mM Ca^2+^/1 mM Mg^2+^.

To more closely approximate physiologically relevant conditions, meEPSCs were recorded at an elevated temperature (30–34°C). Mossy fibers were stimulated using constant voltage pulses (80–100 μs; 20–48 V) delivered through a glass electrode filled with extracellular solution positioned ∼100–200 μm from the recorded granule cell. The criteria for minimal stimulation included an initial ∼30% failure rate during repeated single stimuli at 0.25 Hz and invariant EPSC latency and amplitude with increased stimulus intensity. The mean voltage of the threshold stimulus was 32.7 V for wild-type cells and 34.2 V for *Cln3* ^Δ*ex1–6*^ cells. For each cell, trains of five stimuli (100 Hz, ∼2 V above threshold) were delivered at 3-s intervals and meEPSCs recorded at –70 mV in both “high” and “low” extracellular Ca^2+^ (2 mM Ca^2+^/1 mM Mg^2+^ and 1 mM Ca^2+^/2 mM Mg^2+^). In each case the amplitudes of evoked currents were normalized to the mean amplitude of the first response (meEPSC_1_) in 2 mM Ca^2+^/1 mM Mg^2+^_._

### Transmission electron microscopy

Sagittal slices (200 μm) of cerebellar vermis were prepared from six P13 C57BL/6 mice and three age-matched *Cln3* ^Δ*ex1–6*^ mice, as described above. Slices were cut in slicing solution, immediately transferred into 4% paraformaldehyde and 0.5% glutaraldehyde, and left overnight at 4°C. Following primary fixation, the tissue was washed and osmicated for 1 h at 4°C in 1% OsO_4_ in 0.1 M phosphate buffer, enblocked, stained in 2.0% uranyl acetate buffer for 30 min at 4°C, dehydrated in ethanols, cleared in propylene oxide, and embedded in Araldite. Sections of 70–80 nm in thickness were made. These were collected on copper mesh grids, counterstained with lead citrate, and viewed in a JEOL 1010 electron microscope.

Mossy fiber axons were identified by their structural characteristics (Xu-Friedman and Regehr, 2003). Release sites were identified by the presence of a presynaptic cluster of vesicles close to the membrane, active zone material and a postsynaptic density. Electron micrographs were analyzed by individuals blinded to the genotype and quantified using ImageJ software (v1.46; https://imagej.nih.gov/ij/). To evaluate the density of vesicles in each terminal, a grid composed of multiple squares (each with an area 0.1 μm^2^) was overlaid on the image. We counted the number of vesicles (of ∼30 nm in diameter) within each square. Squares containing organelles, or those containing the border of the mossy fiber terminal were excluded from analysis. Vesicles were considered to be proximal to the release site if they were <100 nm from the presynaptic membrane of an active zone. The active zone vesicle density was then calculated as the number of vesicles per 50 nm of active zone length. As accurate identification of docked vesicles is demanding, even in much thinner slices than used here (Molnár et al., 2016), we opted to count those within one vesicle radius of the active zone and term them “membrane adjacent” vesicles.

### Statistical analysis

Summary data are presented in the text as mean ± SEM from *n* cells (or mossy fiber terminals). Comparisons involving two datasets only were performed using a Wilcoxon rank sum test. For the comparison of paired-pulse ratios (PPRs) at different frequencies and analysis of short-term plasticity, we used two- and three-way repeated measures ANOVA. For EM data, nested analysis was performed using a likelihood ratio test comparing two linear mixed-effect models (Bates et al., 2015). Exact *p* values are presented to two significant figures, except when *p* < 0.0001. Differences were considered significant at *p* < 0.05. Statistical tests were performed using R (version 3.3.2; the R Foundation for Statistical Computing; http://www.r-project.org/) and R Studio (version 1.1.383; RStudio). No statistical test was used to predetermine sample sizes; these were based on standards of the field.

## Results

### Levels of GluA2 and GluA4 are unaltered in cerebella of *Cln3* ^Δ*ex1–6*^ mice

The increased AMPAR-mediated excitotoxicity seen in dissociated and slice cultures of cerebellum from 8- to 10-d-old *Cln3* ^Δ*ex1–6*^ mice was originally suggested to reflect altered AMPAR trafficking, and a possible increase in the number of GluA2-lacking CP-AMPARs (Kovács et al., 2006). However, the same authors later described an increase in GluA2 protein in the cerebellum of one-month-old *Cln3* ^Δ*ex1–6*^ mice (Kovács et al., 2015). To investigate possible AMPAR subunit changes, we initially measured protein levels for GluA2 and GluA4 in cerebellum from wild-type and *Cln3* ^Δ*ex1–6*^ mice in the second postnatal week, around the age when the first structural and functional defects are observed in *Cln3* ^Δ*ex1–6*^ mice (Weimer et al., 2009).

**Figure 1.**
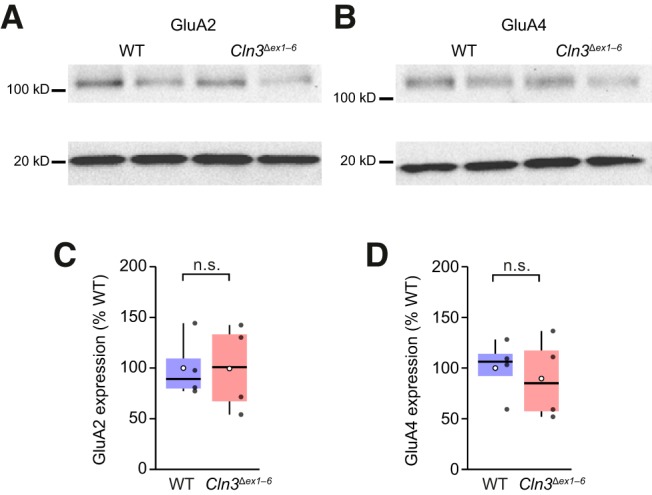
GluA2 and GluA4 expression is unaltered in cerebellum of *Cln3* ^Δ*ex1–6*^ mice. ***A***, Representative western blots comparing the expression of GluA2 in cerebellar lysates from wild-type (WT) and *Cln3* ^Δ*ex1–6*^ mice. Each lane uses pooled tissue from three littermate mice. Upper bands (near 100 kDa) show the labeling for GluA2. Lower bands (at 20 kDa) show the corresponding labeling for cofilin. ***B***, Same as ***A*** but for GluA4. ***C***, Pooled data for GluA2 expression normalized to mean WT expression. Box-and-whisker plots indicate the median value (black line), the 25–75th percentiles (box), and the 10–90th percentiles (whiskers); filled black circles are data from individual cells and open circles indicate means. ***D***, Same as ***C*** but for GluA4 (n.s., non-significant; Wilcoxon rank sum test).

We prepared cerebellar tissue lysate from 12 wild-type and 12 *Cln3* ^Δ*ex1–6*^ mice (P14–P16). For each group, four samples were generated by pooling tissue from three littermate mice. All eight samples were run together and the membrane probed with the relevant antibodies (mouse anti-GluA2, mouse anti-GluA4, rabbit anti-cofilin; see Materials and Methods; Fig. 1*A*,*B*). We found no difference in total protein for either GluA2 (0.29 ± 0.04 for wild type vs 0.29 ± 0.06 for *Cln3* ^Δ*ex1–6*^, normalized to the intensity of the cofilin band; W = 10, *p* = 0.69) or GluA4 (0.33 ± 0.05 vs 0.30 ± 0.07 normalized to the intensity of cofilin; W = 9, *p* = 0.89; Fig. 1*C*,*D*). Similar results were obtained when values were normalized to total protein (data not shown; see Materials and Methods).

### AMPA-evoked currents are unchanged in cultured *Cln3* ^Δ*ex1–6*^ granule cells

**Figure 2.**
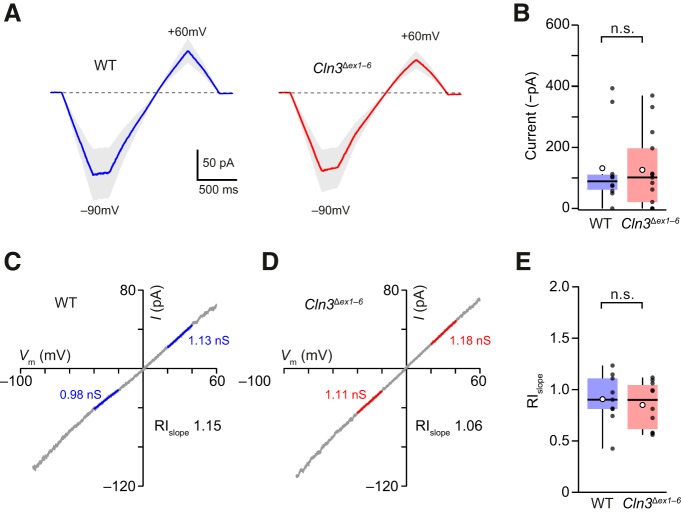
AMPA-evoked whole-cell currents from granule cells of *Cln3* ^Δ*ex1–6*^ mice are similar. ***A***, Global average waveforms of leak-subtracted AMPA-evoked currents (in the presence of 10 μM cyclothiazide) from wild-type (WT) and *Cln3* ^Δ*ex1–6*^ mice (10 and 13 cells, respectively). Shaded areas denote SEM. ***B***, Pooled data showing no change in amplitude (–90 mV) in cells from *Cln3* ^Δ*ex1–6*^ mice. ***C***, Representative *I-V* relationship from a WT cell. Fitted blue lines (from –40 to –20 mV and from +20 to +40 mV) indicate the slope conductances (*G*_slope_) for the negative and positive limbs of the *I-V* relationship. The rectification index (indicated) was calculated as RI_slope_ = *G*_slope_ pos/*G*_slope_ neg. ***D***, Same as ***C*** but for a representative granule cell from a *Cln3* ^Δ*ex1–6*^ mouse. ***E***, Pooled data showing similar rectification in cells from WT and *Cln3* ^Δ*ex1–6*^ mice. Box-and-whisker plots as in Figure 1 (n.s., non-significant; Wilcoxon rank sum test).

To determine whether the magnitude of AMPAR-mediated currents or the prevalence of CP-AMPARs was altered in cerebellar granule cells from *Cln3* ^Δ*ex1–6*^ mice, we first made recordings from cultured neurons and examined whole-cell currents evoked by bath application of AMPA (20 µM). The responses were compared during voltage ramps from –90 to +60 mV, with spermine (500 µM) included in the pipette (intracellular) solution (Fig. 2*A*). As this polyamine blocks CP-AMPARs in a voltage-dependent manner, with pronounced block at depolarized potentials, it allows their presence to be identified from the characteristic inwardly rectifying *I-V* relationship (Bowie and Mayer, 1995; Kamboj et al., 1995; Koh et al., 1995).

We found the mean current amplitude at –90 mV was unaltered in *Cln3* ^Δ*ex1–6*^ cells compared with wild-type (wild-type 131.9 ± 41.4 and *Cln3* ^Δ*ex1–6*^ 126.4 ± 34.7, *n* = 10 and 13, respectively; W = 67, *p* = 0.95; Fig. 2*A*,*B*). This situation persisted when current amplitudes were normalized to the measured cell capacitance. Moreover, the *I-V* plots were similar. Cells from both wild-type and *Cln3* ^Δ*ex1–6*^ mice exhibited near-linear *I-V* relationships (Fig. 2*C*–*E*), with rectification indices (RIs; see Materials and Methods) of 0.91 ± 0.08 and 0.85 ± 0.07, *n* = 9 and 10, respectively (W = 52, *p* = 0.60). This observation suggests that loss of CLN3 does not alter the predominant expression of CI-AMPARs in cultured granule cells.

### mEPSCs and synaptic AMPARs are unaltered in granule cells from *Cln3* ^Δ*ex1–6*^ mice

**Figure 3.**
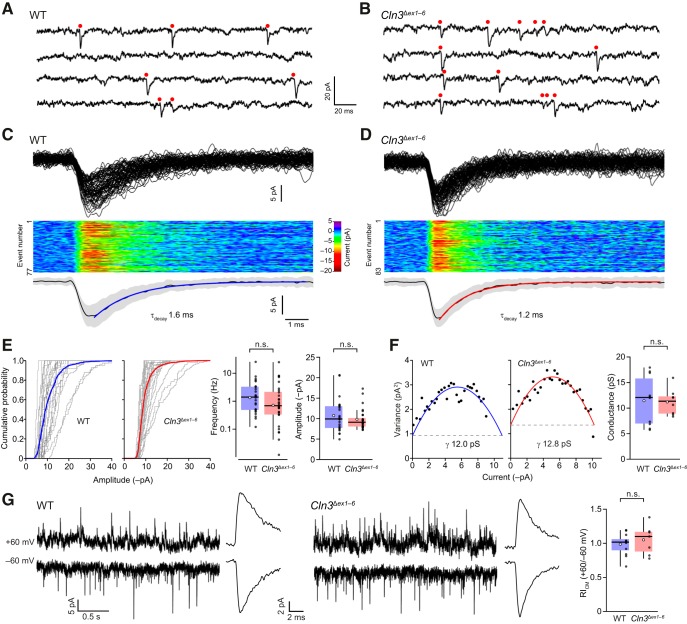
mEPSCs in granule cells from wild-type (WT) and *Cln3* ^Δ*ex1–6*^ mice are indistinguishable. ***A***, Representative recording of mEPSCs from a granule cell in a culture prepared from WT mice (–60 mV). Traces are consecutive and filtered at 1 kHz for display (mEPSCs are indicated by red dots). ***B***, Same as ***A*** but from a granule cell in a culture prepared from *Cln3* ^Δ*ex1–6*^ mice. Scale bars apply to both ***A***, ***B***. ***C***, upper, Individual mEPSCs from the cell in a, aligned at their point of steepest rise. Middle, Color-coded image of all 77 events. Lower, Averaged mEPSC (black trace) with superimposed SEM (gray fill) and exponential fit to the decay (blue line). The time constant (τ_decay_) is indicated. ***D***, Same as ***C*** but for mEPSCs from the *Cln3* ^Δ*ex1–6*^ recording in ***B*** (scale bars apply to both ***C***, ***D***). ***E***, Pooled data showing similar amplitude and frequency of mEPSCs in granule cells from WT and *Cln3* ^Δ*ex1–6*^ mice. Left, Cumulative probability distributions for mEPSC amplitudes. The averaged distributions are shown in bold (WT blue; *Cln3* ^Δ*ex1–6*^ red). Right, Box-and-whisker plots (as in Fig. 1) for mEPSC frequency (log_10_ scale) and amplitude (n.s., non-significant; Wilcoxon rank sum test). ***F***, left, Representative current-variance relationships. The dashed line indicates the background current variance. The single-channel conductance (γ) was calculated from the weighted-mean unitary current estimated from the parabolic fit. Right, Box-and-whisker plots (as in ***E***) showing similar values for conductance. ***G***, Representative recordings from cultured granule cells at −60 and +60 mV with corresponding count-matched averaged mEPSCs (see Materials and Methods). Traces are from a WT cell (left) and a *Cln3* ^Δ*ex1–6*^ cell (right). Far right, Box-and-whisker plots (as in ***E***) showing pooled data for count-matched rectification index (RI_CM_).

We next examined synaptic AMPARs by recording mEPSCs in the presence of TTX (1 µM; Fig. 3*A*–*D*). The amplitude and frequency of mEPSCs at –60 mV was similar in cells cultured from wild-type and *Cln3* ^Δ*ex1–6*^ mice (10.7 ± 0.8 vs 9.8 ± 0.5 pA, W = 374, *p* = 0.49 and 3.1 ± 1.1 vs 2.5 ± 0.9 Hz, W = 404, *p* = 0.22; *n =* 24 and 28 cells, respectively; Fig. 3*C*–*E*).

To determine whether loss of CLN3 led to an alteration in the basic properties of synaptic AMPARs in granule cells, we assessed their kinetics, voltage-dependence and mean single-channel conductance by analyzing synaptic currents. The 10–90% risetime and weighted decay of mEPSCs (see Materials and Methods) did not differ between cells cultured from wild-type and *Cln3* ^Δ*ex1–6*^ mice (0.33 ± 0.02 vs 0.34 ± 0.02 ms, W = 36, *p* = 0.76 and 1.27 ± 0.10 vs 1.42 ± 0.12 ms, W = 33, *p* = 0.57; *n* = 10 and 8 cells). Likewise, we found no difference in the weighted mean single-channel conductance determined using ps-NSFA (see Materials and Methods; 11.5 ± 1.5 vs 11.2 ± 0.9 pS, W = 40, *p* = 1.00; *n* = 10 and 8 cells; Fig. 3*F*) or in mEPSC rectification (RI_CM_ +60/–60 mV; see Materials and Methods; 0.99 ± 0.05 vs 1.05 ± 0.08, W = 36, *p* = 0.65; *n* = 12 and 7 cells; Fig. 3*G*). The fact that the mEPSCs remained non-rectifying and their underlying single-channel conductance remained low in *Cln3* ^Δ*ex1–6*^ mice suggests that, in keeping with the data from whole-cell AMPA-evoked currents, CI-AMPARs are the predominant subtype present at granule cell synapses following loss of CLN3.

### Quantal events at mossy fiber-granule cell synapses of *Cln3* ^Δ*ex1–6*^ mice

**Figure 4.**
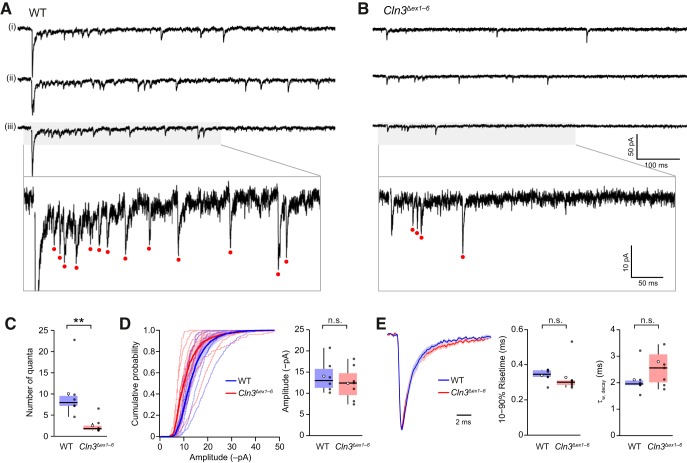
Reduced number of mossy fiber-evoked quantal events in granule cells in acute cerebellar slices from *Cln3* ^Δ*ex1–6*^ mice. ***A***, Representative mossy fiber-evoked responses recorded from a wild-type (WT) granule cell (–70 mV; 0 Ca^2+^/5 mM Sr^2+^). Three consecutive records are shown (i–iii). The region indicated in gray is enlarged in the lower panel to show the detected qEPSCs (red dots). ***B***, Same as ***A*** but in a cell from a *Cln3* ^Δ*ex1–6*^ mouse (scale bars apply to both ***A***, ***B***). ***C***, Box-and-whisker plots (as in Fig. 1) showing the reduced number of discrete quanta evoked in cells from *Cln3* ^Δ*ex1–6*^ mice (**p < 0.01; Wilcoxon rank sum test). ***D***, Cumulative probability distributions for qEPSC amplitudes. Data from each cell are shown together with the averaged distributions in bold (WT, blue; *Cln3* ^Δ*ex1–6*^, red). Shaded areas denote SEM. Right, Box-and-whisker plots (as in ***C***) showing unaltered qEPSC amplitude in cells from *Cln3* ^Δ*ex1–6*^ mice. ***E***, Superimposed normalized global average qEPSC waveforms from six WT and seven *Cln3* ^Δ*ex1–6*^ cells show no differences. Shaded areas denote SEM. Right, Box-and-whisker plots (as in ***C***) for qEPSC 10–90% risetime and τ_w, decay_ (n.s., non-significant; Wilcoxon rank sum test).

To investigate transmission at mossy fiber to granule cell synapses formed *in vivo*, we next moved to acute cerebellar slices. As spontaneous mEPSCs occurred only at low frequency, we initially examined quantal events (qEPSCs) in response to mossy fiber stimulation. We made recordings in the presence of 5 mM extracellular SrCl_2_ to trigger the asynchronous release of transmitter such that individual quanta could be identified (Fig. 4*A*,*B*). This approach allowed us to measure both the size and the number of quanta released per stimulus.

Unexpectedly, in slices from *Cln3* ^Δ^*^ex1–6^*mice, each mossy fiber stimulation evoked a smaller initial EPSC and far fewer discrete qEPSCs than in wild type (initial amplitude reduced from –52.3 ± 6.9 pA to –21.2 ± 6.3 pA, *n* = 6 and 7, W = 4, *p* = 0.014 and number of quantal events reduced from 10.0 ± 2.6-2.5 ± 0.7; W = 41, *p* = 0.0023; Fig. 4*C*). Of note, in slices from wild-type mice no “failures” (sweeps in which no response was evoked) were seen, but in slices from *Cln3* ^Δ^*^ex1–6^*mice, the average failure rate was ∼10% (range 0–23.3%). In slices from *Cln3* ^Δ^*^ex1–6^*mice the amplitude of qEPSCs was similar to wild-type (14.0 ± 1.6 vs 12.4 ± 1.5 pA, *n* = 6 and 7; W = 24, *p* = 0.73; Fig. 4*D*), and both the 10–90% risetime (RT_10–90%_; 0.34 ± 0.01 vs 0.33 ± 0.03 ms; W = 30.5, *p* = 0.20) and weighted decay time (τ_w, decay_; 2.11 ± 0.23 vs 2.79 ± 0.44 ms; W = 13, *p* = 0.29) of qEPSCs remained unchanged (Fig. 4*E*). These results demonstrate no change in postsynaptic responsiveness at mossy fiber synapses of *Cln3* ^Δ*ex1–6*^ mice, but the activation of fewer mossy fibers or a potential reduction in the probability of transmitter release.

### Unaltered paired-pulse depression of eEPSCs in *Cln3* ^Δ*ex1–6*^ granule cells

Mossy fiber-granule cell synapses are known to sustain high bandwidth transmission, but the majority show an initial short-term depression during high frequency stimulation (Nieus et al., 2006; Saviane and Silver, 2006; Chabrol et al., 2015). Although the reduced number of qEPSCs in slices from *Cln3* ^Δ^*^ex1–6^*mice could be consistent with a decrease in release probability in 0 Ca^2+^/5 mM Sr^2+^, this was not evident when we examined eEPSCs in 2 mM Ca^2+^. Responses to paired stimuli at 5, 10, 20 and 100 Hz showed no difference in PPR. For example, at 100 Hz, the PPR indicated similar magnitude of depression (0.41 ± 0.13 and 0.30 ± 0.05 for wild-type and *Cln3* ^Δ*ex1–6*^ cells; *n* = 5 and 4, respectively; W = 10, *p* = 1.00, Wilcoxon rank sum test). Across the frequency range examined, two-way RM ANOVA showed an effect of inter stimulus interval (*F*_(3,21)_ = 16.88, *p* < 0.0001), no effect of genotype (*F*_(1,7)_ = 0.24, *p* = 0.64), and no interaction (*F*_(3,21)_ = 0.80, *p* = 0.51). As both qEPSCs and eEPSCs were recorded under “non-physiological” conditions, we next chose to examine synaptic transmission at near-physiological temperature, and in 1 mM extracellular Ca^2+^, a concentration thought likely to approximate more closely the situation *in vivo* (Borst, 2010).

### Altered short-term plasticity of meEPSCs in *Cln3* ^Δ*ex1–6*^ mice in reduced [Ca^2+^]_o_

**Figure 5.**
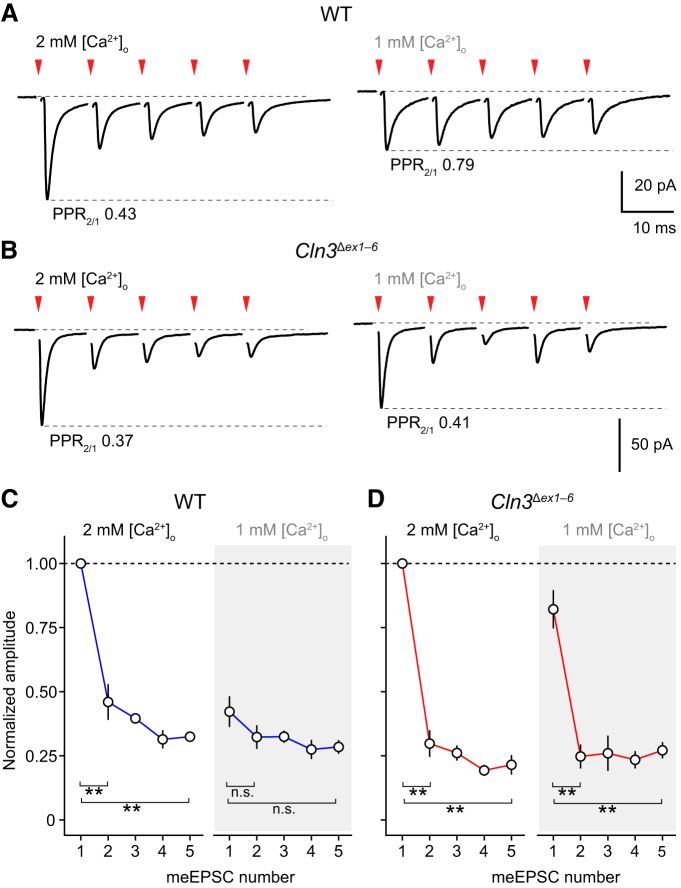
meEPSCs in granule cells in slices from wild-type (WT) and *Cln3* ^Δ*ex1–6*^ mice exhibit different patterns of short-term plasticity in low extracellular Ca^2+^. ***A***, Averaged meEPSCs from a representative WT granule cell evoked during a five-pulse 100-Hz train in the presence of 2 and 1 mM extracellular Ca^2+^ (–70 mV; 428 and 110 sweeps, respectively). Red arrowheads indicate timing of stimuli (stimulus artifacts are blanked). PPRs (meEPSC_2_/meEPSC_1_) are indicated as PPR_2/1_. ***B***, Same as ***A*** but for a representative *Cln3* ^Δ*ex1–6*^ granule cell (197 and 111 sweeps). ***C***, Plots showing normalized meEPSC amplitude in WT granule cells during five-pulse trains in 2 and 1 mM Ca^2+^. Symbols denote mean and error bars SEM. ***D***, Plots (as in ***C***) but for *Cln3* ^Δ*ex1–6*^ granule cells (**p < 0.01 and n.s., non-significant; paired Wilcoxon rank sum test with Holm’s sequential Bonferroni correction for multiple comparisons).

We examined meEPSCs in response to brief trains of high frequency mossy fiber stimulation (five stimuli at 100 Hz) at 30–34°C in both “normal” and reduced extracellular Ca^2+^ (2 mM Ca^2+^/1 mM Mg^2+^ and 1 mM Ca^2+^/2 mM Mg^2+^; Fig. 5*A*,*B*). For each cell (six wild type and six *Cln3* ^Δ*ex1–6*^), meEPSC amplitudes were normalized to that of the first event in 2 mM extracellular Ca^2+^. In both groups of mice, we observed a wide range of amplitudes for the first meEPSC (peak conductance of 0.56–2.91 nS for wild-type and 0.33–1.56 nS for *Cln3* ^Δ*ex1–6*^), within the wide range (0.11–3.33 nS) reported by Chabrol et al. (2015) for different mossy fiber input pathways. In 2 mM Ca^2+^, meEPSCs in granule cells from wild-type mice exhibited short-term depression (meEPSC_2_/meEPSC_1_ was 0.46 ± 0.07; W = 36, *p* = 0.0028). When the same cells were recorded in 1 mM extracellular Ca^2+^, there was no depression (meEPSC_2_/meEPSC_1_ was 0.81 ± 0.12; W = 27, *p* = 0.18; Fig. 5*A*). However, for *Cln3* ^Δ*ex1–6*^ cells, paired-pulse depression was seen in both 2 and 1 mM extracellular Ca^2+^ (0.30 ± 0.05 and 0.31 ± 0.06, respectively; both W = 36, *p* = 0.0028 and *p* = 0.0022). A three-way repeated measures ANOVA was run to examine the effect of stimulus number, extracellular Ca^2+^ concentration and genotype on meEPSC amplitude (normalized to meEPSC_1_ in 2 mM Ca^2+^). There was a significant three-way interaction, *F*_(4,80)_ = 3.67, *p* = 0.0085. Thus, the effect of lowering extracellular Ca^2+^ on the meEPSC amplitudes during short trains was affected by deletion of CLN3. Overall, these results suggest altered release dynamics in *Cln3* ^Δ*ex1–6*^ mice, that are revealed in conditions of reduced extracellular Ca^2+^. Of note, the mean amplitude of meEPSC_1_ in 2 mM Ca^2+^ did not differ between genotypes (77.3 ± 26.0 and 69.7 ± 12.0 pA; W = 14, *p* = 0.59), but amplitudes of meEPSC_1_ in 1 mM Ca^2+^ (normalized to those of meEPSC_1_ in 2 mM Ca^2+^) were different (0.42 ± 0.06 and 0.82 ± 0.07 in wild type and *Cln3* ^Δ*ex1–6*^, respectively; W = 2, *p* = 0.0087).

### Structural changes at mossy fiber-granule cell synapses in *Cln3* ^Δ*ex1–6*^ mice

We next used 2D transmission electron microscopy to compare mossy fiber to granule cell synapses from *Cln3* ^Δ*ex1–6*^ and wild-type mice (P13). Mossy fiber rosettes were identified from their characteristic size and appearance (many small vesicles, and a large number of mitochondria; Xu-Friedman and Regehr, 2003; Rothman et al., 2016), and the fact that the mossy fiber makes contact with a large number of granule cell dendrites.

**Figure 6.**
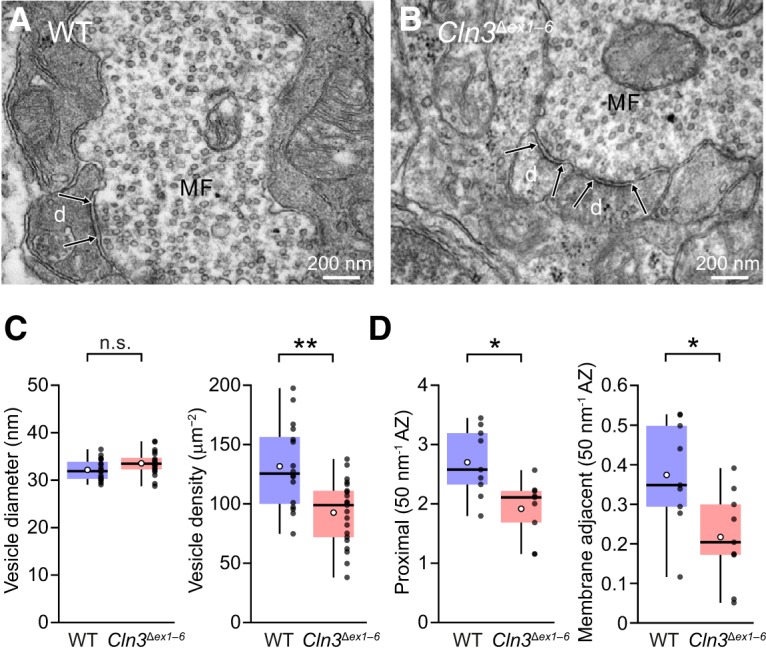
Reduced vesicle density in mossy fiber terminals of *Cln3* ^Δ*ex1–6*^ mice. ***A***, Representative electron micrograph showing a wild-type (WT) mossy fiber terminal (MF) making a synaptic contact (delineated by arrows) with a granule cell dendrite (d). ***B***, Same as ***A*** but from a *Cln3* ^Δ*ex1–6*^ mouse. ***C***, Box-and-whisker plots (as in Fig. 1) showing the unaltered vesicle diameter and the reduced vesicle density. ***D***, Box-and-whisker plots (as in Fig. 1) showing the reduced number of vesicles proximal to active zones (AZ) and reduced number of membrane adjacent vesicles in MF terminals from *Cln3* ^Δ*ex1–6*^ mice (**p < 0.01, *p < 0.05; Wilcoxon rank sum test).

Initial examination revealed no striking gross anatomic differences between *Cln3* ^Δ*ex1–6*^ and wild-type synapses (Fig. 6*A*,*B*). The average vesicle diameter was unchanged in *Cln3* ^Δ*ex1–6*^ compared to wild type (mean vesicle diameter per mossy fiber terminal 33.5 ± 0.5 vs 32.2 ± 0.5 nm, *n =* 20 and 19 terminals from three mice each; W = 130, *p* = 0.094; Fig. 6*C*). We observed a high average density of vesicles within each mossy fiber terminal, comparable to the values of 118–170 μm^−2^ reported by Rothman et al. (2016). However, the average density of vesicles per mossy fiber terminal was decreased in *Cln3* ^Δ*ex1–6*^ mice, from 131.7 ± 8.9 to 92.6 ± 6.0 μm^−2^ (*n* = 16 and 21 terminals; W = 267, *p* = 0.0025; Fig. 6*C*). Additionally, when we determined the number of vesicles proximal to each active zone (within 100 nm), the average number per 50-nm length of active zone was reduced by ∼30% (from 2.70 ± 0.19 to 1.92 ± 0.16, *n* = 9 terminals in each of three mice; W = 69, *p* = 0.013; Fig. 6*D*). When we considered only membrane adjacent vesicles (those within one vesicle radius of the presynaptic membrane), the number was reduced by ∼40% (from 1.24 ± 0.16 to 0.71 ± 0.15 per active zone; W = 67, *p* = 0.022 and from 0.37 ± 0.04 to 0.22 ± 0.04 per 50 nm of active zone; W = 65, *p* = 0.034; Fig. 6*D*). Of note, use of nested analysis (see Materials and Methods), rather than average measures per terminal, did not qualitatively alter the outcome. Thus, while vesicle diameter was unchanged, the overall vesicle density per terminal was slightly decreased, as was the average number of vesicles proximal to active zones and the number of membrane adjacent vesicles.

## Discussion

We have examined granule cell AMPARs and cerebellar mossy fiber to granule cell synapses in the *Cln3* ^Δ*ex1–6*^ mouse, a widely used model of juvenile Batten disease. Our main findings are as follows: First, GluA2 and GluA4 expression in cerebellar tissue from *Cln3* ^Δ*ex1–6*^ mice is unaltered. Second, AMPA-evoked currents in granule cells cultured from wild-type and *Cln3* ^Δ*ex1–6*^ mice are not different. Third, the properties of synaptic AMPARs, their kinetics, voltage-dependence, and single-channel conductance, are unaltered. Fourth, loss of CLN3 leads to altered short-term plasticity in conditions of reduced extracellular Ca^2+^. Fifth, in mossy fiber terminal from *Cln3* ^Δ*ex1–6*^ mice the density of synaptic vesicles and their proximity to active zones is reduced. Thus, our experiments reveal unanticipated presynaptic changes but no evidence for altered postsynaptic AMPARs.

### Changes in synaptic transmission occur early in *Cln3* ^Δ*ex1–6*^ mice

Although the original studies of *Cln3* ^Δ*ex1–6*^ mice reported accumulation of lysosomal storage material at approximately three months of age (Mitchison et al., 1999; Seigel et al., 2002) the mice were thought to lack clinical symptoms, even at 12 months (Mitchison et al., 1999). Subsequent studies identified deficits in motor coordination as early as P14 (Kovács et al., 2006), which were preceded by thinning of the cerebellar granule cell layer and Purkinje cell loss (Weimer et al., 2009). Our results suggest that there are indeed early changes in synaptic transmission in the cerebellum of *Cln3* ^Δ*ex1–6*^ mice (P10–P15).

Our experiments do not allow us to conclude whether the observed changes are a direct consequence of CLN3 loss or represent secondary effects. In this regard, it is of note that extensive changes in gene expression and protein levels occur in *Cln3* ^Δ*ex1–6*^ mice (Brooks et al., 2003; Llavero Hurtado et al., 2017), potentially disrupting multiple neuronal pathways. Nevertheless, our findings in a mouse model of juvenile CLN3 disease complement molecular, structural, and functional studies in various animal models of infantile CLN1 disease (Virmani et al., 2005; Kim et al., 2008; Kielar et al., 2009), late infantile CLN6 disease (Kielar et al., 2009), congenital CLN10 disease (Koch et al., 2011), and late infantile CLN5 disease (Amorim et al., 2015), and suggest that early synaptic alteration is a characteristic feature of NCLs.

### No change in the rectification of AMPARs in *Cln3* ^Δ*ex1–6*^ granule cells

Previous studies reported increased AMPA-mediated neurotoxicity in dissociated granule cells and organotypic cultured cerebellar slices from one-week-old *Cln3* ^Δ*ex1–6*^ mice (Kovács et al., 2006), and improved motor skills in one- to seven-month-old mice following AMPAR blockade (Kovács and Pearce, 2008; Kovács et al., 2011). These authors proposed an increase in the number of CP-AMPARs in *Cln3* ^Δ*ex1–6*^ cerebellar granule cells, and abnormally increased AMPA receptor-mediated neurotransmission in the cerebellum. More recently, the same authors reported an increase in both total and surface GluA2 in acute cerebellar slices from one-month-old *Cln3* ^Δ*ex1–6*^ mice, and proposed a decrease in the number of CP-AMPARs (Kovács et al., 2015). Our biochemical analysis and patch-clamp recordings do not support either of these proposals. We found no difference in the levels of GluA2 or GluA4 protein in cerebellar lysates of wild-type and *Cln3* ^Δ*ex1–6*^ mice. Importantly, we found the magnitude and *I-V* relationships of AMPAR-mediated currents obtained in the presence of intracellular spermine to be similar in cultured cerebellar granule cells from wild-type and *Cln3* ^Δ*ex1–6*^ mice. In both groups of mice, *I-V* relationships were linear, a feature characteristic of GluA2-containing calcium-impermeable AMPARs. Given that mEPSCs in *Cln3* ^Δ*ex1–6*^ cells exhibited no detectable alteration in amplitude, rise time, decay time, rectification properties or underlying mean single-channel conductance, it seems highly likely that the number and composition of AMPARs at synapses was also unchanged. The reason for these disparities is unclear, but it should be noted that our studies were conducted using mice on a C57BL/6J background, whereas the work of Pearce and colleagues used mice on a 129S6/SvEv background. Importantly, while there are some background-specific differences in motor phenotype of these *Cln3* ^Δ*ex1–6*^ strains, both exhibit clear motor deficits (Kovács and Pearce, 2015).

### Presynaptic changes at mossy fiber-granule cell synapses in *Cln3^Δex1–6^* mice

As with mEPSCs in cultured granule cells, the amplitude and kinetics of qEPSCs evoked at mossy fiber to granule cell synapses (in the presence of Sr^2+^) were unaffected by loss of CLN3. However, we found a marked decrease in the number of quanta released per stimulus in *Cln3* ^Δ*ex1–6*^ mice. This could indicate a reduction in the probability of release or simply the activation of fewer mossy fibers. Intriguingly, a recent report described increased hippocampal field excitatory post-synaptic potentials in *Cln3* ^Δ^*^ex7/8^*mice and suggested increased axonal excitability at the earliest age studied (one month; Burkovetskaya et al., 2017), tending to argue against the second of these possibilities. In a separate set of experiments in 2 mM Ca^2+^, we found the PPR of eEPSCs was not affected by loss of CLN3, suggesting no change in release probability. Thus, the effect of CLN3 loss may depend on the extracellular Ca^2+^ concentration.

In both wild-type and *Cln3* ^Δ*ex1–6*^ slices, we observed depression of eEPSC amplitudes during short trains of mossy fiber stimulation in the presence of standard extracellular divalent cations (2 mM Ca^2+^/1 mM Mg^2+^). Surprisingly, when we reduced release probability by lowering extracellular Ca^2+^ (1 mM Ca^2+^/2 mM Mg^2+^), we observed loss of depression in wild-type cells (Nieus et al., 2006; Saviane and Silver, 2006) but not in *Cln3* ^Δ*ex1–6*^ cells. The fact that the loss of CLN3 appeared to have a functional impact on transmission only when extracellular Ca^2+^ was reduced suggests the possibility of an alteration in Ca^2+^ handling or sensing. Recent studies have indeed suggested that in both neurons (Warnock et al., 2013) and neuronal progenitor cells (Chandrachud et al., 2015) calcium handling is disrupted following loss of CLN3. This has been shown to result in the aberrant elevation of intracellular Ca^2+^ following K^+^-induced depolarization or moderate inhibition of the sarco/endoplasmic reticulum Ca^2+^-ATPase by thapsigargin. Whether altered Ca^2+^ handling in mossy fiber terminals could account for the differences in short term plasticity between *Cln3* ^Δ*ex1–6*^ and wild-type mice is unclear.

### Ultrastructural changes at mossy fiber terminals in *Cln3^Δex1–^* ^6^ mice

Our 2D EM analyses revealed presynaptic structural changes in *Cln3* ^Δ*ex1–6*^ mice, including a decrease in the vesicle density per mossy fiber terminal, a decrease in the number of vesicles proximal to active zones, and a decrease in membrane adjacent vesicles. Interestingly, broadly similar findings have been described in a different NCL. A reduction in vesicle number has been seen in cortical neurons from palmitoyl-protein thioesterase-1 knock-out mice (*Ppt1* ^–/–^), a model of infantile CLN1 disease (Virmani et al., 2005; Kim et al., 2008). This effect was linked with persistent membrane association of palmitoylated synaptic vesicle proteins preventing endocytosis. Conversely, in cathepsin D knock-out mice (*Ctsd* ^–/–^), a model of congenital CLN10 disease, there is a reported increase at hippocampal CA1 synapses in the total vesicle number and in the number of docked vesicles (Koch et al., 2011). Thus, changes in the presynaptic vesicle pool may be a common feature of multiple NCLs. How, or if, the reduction we observe in synaptic vesicles of *Cln3* ^Δ*ex1–6*^ mice relates to previously described changes in intracellular vesicular trafficking of CLN3-deficient cells (Fossale et al., 2004; Metcalf et al., 2008; Tecedor et al., 2013; Wavre-Shapton et al., 2015) remains to be determined. However, it is possible that the reduced vesicle numbers constitute a compensatory mechanism to overcome the tendency toward elevated release under physiologic conditions. This idea follows from our observation that in 1 mM Ca^2+^ normalized amplitudes of meEPSC in *Cln3* ^Δ*ex1–6*^ mice were greater than those of wild-type mice. Of note, the reduced Ca^2+^ recordings are likely to reflect more accurately the situation *in vivo*, where the concentration of extracellular Ca^2+^ is thought to be closer to 1 rather than 2 mM (Borst, 2010).

Very recently, a paper was published which described disruption of supraspinal synaptic transmission in the *Cln3* ^Δ*ex1–6*^ mouse due to impaired presynaptic release, and proposed this as a causative mechanism in juvenile Batten disease (Grünewald et al., 2017). CLN3 loss was found to impair inhibitory PSCs or inhibitory synaptic transmission and to cause loss of GABAergic interneurons, in amygdala, hippocampus, and cerebellum. In addition, the authors reported a reduction in the amplitude of eEPSCs in both principal neurons of the lateral amygdala and granule cells of the dentate gyrus, no change in the amplitude of mEPSCs or spontaneous EPSCs, but a reduction in their frequency. Paired-pulse facilitation during stimulation of the lateral perforant path was also reduced. Overall, the findings were interpreted as reduction of excitatory and inhibitory inputs. Our results echo these observations in identifying presynaptic changes in *Cln3* ^Δ*ex1–6*^ mice.

Irrespective of the precise mechanism underlying synaptic changes in *Cln3* ^Δ*ex1–6*^ mice (Cárcel-Trullols et al., 2015; Grünewald et al., 2017), our observations are potentially important in understanding the locus of early changes in juvenile Batten disease. While the recent study of Grünewald et al. (2017) examined synaptic function in symptomatic (14-month-old) *Cln3* ^Δ*ex1–6*^ mice, the synaptic changes we observed in two-week-old mice occurred in the presymptomatic phase of the disease, and are thus likely to reflect the initial causative changes. Previous functional studies focused primarily on an apparent selective increase in AMPAR function in cerebellar granule cells of *Cln3* ^Δ*ex1–6*^ mice, and proposed a block of AMPARs as a potential therapeutic approach (Kovács et al., 2011). Importantly, our results argue strongly against any early change in postsynaptic AMPARs.

## Synthesis

Reviewing Editor: Zoltan Nusser, Institute of Experimental Medicine Decisions are customarily a result of the Reviewing Editor and the peer reviewers coming together and discussing their recommendations until a consensus is reached. When revisions are invited, a fact-based synthesis statement explaining their decision and outlining what is needed to prepare a revision will be listed below. The following reviewer(s) agreed to reveal their identity: Skyler Jackman, Jason Rothman

In this manuscript the authors investigate synaptic transmission in Cln3 knockout mice. They use cultured cerebellar granule cells and cerebellar slices to perform experiments aimed at determining whether synaptic deficits described in the literature result from either presynaptic or postsynaptic changes. They conclude that the AMPA receptor expression levels and receptor composition are unchanged in the cerebellum. They see no change in mEPSC amplitude, kinetics or frequency in cerebellar slices. However, when they stimulate mossy fibre inputs onto granule cells in the presence of strontium they report smaller evoked EPSCs and less asynchronous (total) release. Using minimal stimulation experiments in acute slices, they conclude that Cln3 knockout results in changed presynaptic release properties.

The reviewers raised a number of points (see below), out which I would like to highlight the importance of three. First, the authors should provide more information (data) regarding the way the minimal stimulation experiments were conducted. They should present stimulus intensity-EPSC amplitude plots in control and KO animals. These experiments are important in light of potential alterations in the excitability of the axons in KO mice (Burkovetskaya 2016). The minimal stimulation experiments should be repeated in normal ACSF (2 mM Ca2+ / 1 mM Mg2+), and show the input size vs. stimulus intensity relationships, and demonstrate a clear response threshold for a large number of cells in both genotypes.

The authors describe an unexpected alteration in the short-term plasticity of EPSCs in low Ca2+. The authors should carry out more experiments to reveal the reason why there is larger depression in the KO mice in which the authors claim that there is a reduced release probability. They should also discuss this somewhat contradictory result.

The analysis of the EM images should have been performed in a ‘blind’ manner. The authors should re-analyse the images by coding the images, performing the analysis by an independent expert on the ‘blinded images’ and revealing the code after the analysis.

Additional points:

The introduction states “to date there have been no investigations of excitatory synaptic currents in mouse models of this condition.” This is no longer the case, with the publication of “Defective synaptic transmission causes disease signs in a mouse model of juvenile neuronal ceroid lipofuscinosis” (Grunewald et al 2017, eLife). The authors should cite and discuss this paper. They should also devote more comparison to results from Burkovetskaya 2016.

Could the authors describe their methods for bath applying AMPA in more detail? I worry whether 10 uM cyclothiazide might be insufficient to block desensitization for long applications.

The authors end the introduction with the following “Our results suggest that loss of CLN3 does not lead to a change in postsynaptic AMPARs, but to altered transmitter release that may be related to changes in Ca2+ handling or sensing”. No evidence is presented regarding calcium handling or sensing, so this sentence feels like speculation based on previous work. At most their results are consistent with changes to presynaptic release properties.

Methods: General comments

1. The authors do not explain how they know the cells they recorded from are granule cells. The authors should give values for cell capacitance and access resistance. They could also verify these values were not statistically different for the WT and knockout mice populations.

2. It is not clear why the authors use both cultures and slices in this study.

Methods: mEPSCs (p. 5)

1. The reference to NeuroMatic website should be moved forward to this section.

2. The authors should mention the threshold used during event detection.

3. Peak-scaled non-stationary fluctuation analysis (ps-NSFA). The authors should reference this analysis technique.

Methods: qEPSCs (p. 6)

1. Are not mEPSCs and qEPSCs the same?

2. “To record quantal EPSCs (qEPSCs), the standard extracellular solution was replaced with a Ca2+-free solution containing 5 mM SrCl2”. The authors should reference this technique. Moreover, according to Xu-Friedman and Regehr (2000), “Strontium is capable of supporting synaptic transmission, but release is dramatically different from that evoked in calcium.” The authors should explain why this does not affect the conclusions of their study.

3. How did the authors determine what stimulus strength to use in these quantal experiments? (see main comments!) Especially given the difference in failure rates between WT and knockout mice.

Methods: meEPSCs (p. 7)

1. “Minimally-evoked EPSCs (meEPSCs) were recorded at an elevated temperature (30-34 {degree sign}C).” It is not explained in manuscript why these recordings were made at higher temperature when all the other recordings were made at room temperature.

Methods: EM (p. 7)

1. “The number of vesicles (~ 50 nm radius) within each square was counted.” It is not clear what 50 nm radius refers to.

2. “Docked vesicles were considered to be those within 1 vesicle radius of the active zone.” The authors could state what this radius value is. Docked vesicles are usually defined as vesicles that are in direct contact with the presynaptic AZ membrane. The authors should analyse their images in light of this criterion.

Methods: Statistical Analysis (p. 8)

1. “No blinding or randomization was used.” Would not blinding been a useful approach when measuring vesicle densities of WT and knockout mice?

Results: General Comments

1. The authors make no comparison of their results to previous publications. For example, GluA2 and GluA4 expression, EPSC amplitude, rise and decay times, vesicle densities, for WT and possibly knockout mice.

Results: Levels of GluA2 and GluA4 (p. 8)

1. “We found no difference in total protein for either GluA2 ... or GluA4...” The results in this section are hardly mentioned in Abstract and Discussion. Perhaps the authors realize these results are weaker than their electrophysiology results. Moreover, there is no comparison of these results to those of previous studies which did find differences in GluA2 expression, for example Kovacs et al. 2015, Figure 1B. Nor a discussion of this discrepancy.

Results: AMPA-evoked currents (p. 9)

1. This section discusses results in context of CP-AMPARs but the last summary sentence switches context to CI-AMPARs. It would be clearer to discuss one or other or both throughout.

2. Why did the authors choose -90 mV to compute current values? This seems non-physiological for AMPAR activation.

Results: mEPSCs (p. 9-10)

1. The first sentence could also state recordings where made from cultures.

2. The amplitude and frequency of mEPSCs were computed for 24 WT cells and 28 Cln3-/- cells, but then rise and decay times were computed for only 10 and 8 cells, respectively. Why did the authors not use all the cells in the latter analysis?

Results: Quantal release (p. 10)

1. “To investigate transmission at mossy fiber to granule cell synapses formed in vivo we next moved to acute cerebellar slices.” Electrophysiology in slices is considered in vitro so this sentence sounds incorrect.

2. “Of note, in slices from wild-type mice no ‘failures’ (sweeps in which no response was evoked) were seen, but in slices from Cln3-/- mice the average failure rate was ~10% (range 0 - 23.3%).” How the authors determined what stimulus strength to use in these experiments was not mentioned in Methods. One could ask if these results are due to a difference in stimulus strength. (see main comments)

3. The weighted decay times of the qEPSCs are twice as long as those of the mEPSCs (the rise times are about the same). Do the authors have an explanation for this discrepancy?

Results: Paired-Pulse Depression (p. 10-11)

1. “...two-way RM ANOVA showed ... no interaction (F3,21 = 0.80, p = 0.51).” What measures was the interaction computed for?

Results: Short-term Plasticity (p. 11)

1. “...normalized amplitude of meEPSC1 in 1 mM Ca2+ were different...” The authors may want to explain what normalization was used.

Discussion: No change in the rectification of AMPARs (p. 13)

1. This finding of the authors is a big discrepancy with the previous literature, which predominantly claim there is a change in post-synaptic AMPARs. The discussion here does not address this adequately. There is only one sentence that attempts to: “Our studies were carried out using mice on a C57BL/6J background (Wavre-Shapton et al., 2015), whereas the earlier work used mice on 129S6/SvEv background.” But this sentence is vague.

Discussion: Ultrastructural Changes (p. 14)

1. “However, it is possible that the reduced vesicle numbers constitute a compensatory mechanism to overcome the tendency towards elevated release under physiological conditions. This idea follows from our observation that in 1 mM Ca2+ glutamate release in Cln3-/- mice was greater than expected, as eEPSC amplitude was not reduced from that seen in 2 mM Ca2+.” The reasoning of last sentence is not clear, especially the last phrase about 2 mM Ca2+. The authors should attempt to rephrase. One's first assumption is that, since Cln3-/- mice have less vesicles, they should have reduced vesicle release compared to WT. For example, one would think a smaller pool of docked vesicles would lead to smaller 1st EPSC, not larger.

Figure 2. “AMPA-evoked whole-cell currents from granule cells of Cln3-/- mice are similar.” The authors should include ‘culture’ and ‘WT mice’ in this sentence. Panel a, the authors should state current recordings are in response to voltage ramps.

Figure 4. The authors should state recordings were made in slice tissue. For the knockout-mice data in b, the first EPSC response to external stimulation looks dramatically smaller than that of WT response. Was this consistent for all other cells or just this one cell?

Figure 5. The authors should state recordings were made in slice tissue.
